# Secondary Bacterial Infections Associated with Influenza Pandemics

**DOI:** 10.3389/fmicb.2017.01041

**Published:** 2017-06-23

**Authors:** Denise E. Morris, David W. Cleary, Stuart C. Clarke

**Affiliations:** ^1^Infectious Disease Epidemiology Group, Academic Unit of Clinical and Experimental Sciences, Faculty of Medicine, Institute for Life Sciences, University of Southampton, University Hospital Southampton Foundation NHS TrustSouthampton, United Kingdom; ^2^Global Health Research Institute, University of SouthamptonSouthampton, United Kingdom; ^3^NIHR Southampton Respiratory Biomedical Research UnitSouthampton, United Kingdom

**Keywords:** influenza, *Streptococcus pneumoniae*, *Haemophilus influenzae*, *Staphylococcus aureus*, pandemic

## Abstract

Lower and upper respiratory infections are the fourth highest cause of global mortality (Lozano et al., 2012). Epidemic and pandemic outbreaks of respiratory infection are a major medical concern, often causing considerable disease and a high death toll, typically over a relatively short period of time. Influenza is a major cause of epidemic and pandemic infection. Bacterial co/secondary infection further increases morbidity and mortality of influenza infection, with *Streptococcus pneumoniae*, *Haemophilus influenzae*, and *Staphylococcus aureus* reported as the most common causes. With increased antibiotic resistance and vaccine evasion it is important to monitor the epidemiology of pathogens in circulation to inform clinical treatment and development, particularly in the setting of an influenza epidemic/pandemic.

## Introduction

From the Plague of Athens to the present day, infectious disease has beset mankind throughout history. Medical and socio-economic advances have substantially reduced this burden, the eradication of smallpox in 1979 (World Health Organization, 2017) and the remarkable successes against polio and parasitic Guinea worm disease being three examples of an extensive list. Respiratory tract infections, however, continue to be a major cause of morbidity and mortality worldwide (Lozano et al., 2012; Morse et al., 2012; Zumla et al., 2014). When combined, lower and upper respiratory infections are the fourth highest cause of global mortality (Lozano et al., 2012). Epi- and pandemic outbreaks of respiratory infection are a major medical concern, often causing considerable disease and a high death toll, typically over a relatively short period of time. The unpredictable nature of these outbreaks, in terms of their etiology and the reservoirs from which they emerge, the constant emergence of new antigenic variants by mutation, combined with transmission within potentially immunologically naïve populations facilitates the characteristic high proficiency of spread (Morse et al., 2012).

It is well established that both animals and humans can act as reservoirs of infection within which pathogens may adapt and evolve. Examples include *Coxiella burnetii* which typically causes Q fever in cattle, sheep and goats but can also infect humans (Eldin et al., 2017), the plague causing *Yersinia pestis*, infamously transmitted to humans by rats via a flea vector (Yang et al., 2016b), human immunodeficiency virus (HIV) which originated in non-human primates before spreading into the human population (Rupp et al., 2016) and of course the most common example, influenza, which circulates within and between swine, avian and human hosts (amongst others). This cross-species flow can lead to adaptations that result in an increased pathogenicity to susceptible hosts, creating the potential for localized outbreaks or global spread (Murphy, 1998; Karesh et al., 2012; Morse et al., 2012). Important evolutionary modifications can occur during the timespan of an individual infection, permitting new and evolved strains of pathogens to emerge at an increased rate (Karesh et al., 2012). The evolution of pathogens (particularly zoonotic pathogens which account for 60% of human infectious diseases), and development of pandemics and epidemics, can be described in ecological principles whereby changing environmental pressures or opportunities drive a pathogen to exploit new niches or hosts to survive and thrive. This evolution is influenced by a range of anthropogenic factors, which include population expansion, changing land use and habitat destruction, selective pressures of increased antimicrobial usage, vaccination, global trade and travel (Daszak, 2012; Karesh et al., 2012; Morse et al., 2012).

Pandemics are generally viral in cause. This is thought to be due to their high mutation rate, which is particularly true for RNA viruses such as influenza where high nucleotide substitution and poor proof reading leads to the accumulation of errors in newly synthesized RNA strands. Influenza can also undergo re-assortment during mixed infection. These factors can result in divergence of surface antigens, such as haemagglutinin (HA) and neuraminidase (NA), producing strains not recognized by the human immune system and not covered by extant vaccines (Holland et al., 1982; Webster et al., 1992; Chen and Holmes, 2006; Hampson and Mackenzie, 2006; Jones et al., 2008; Taubenberger and Morens, 2008; Dormitzer et al., 2011; Morse et al., 2012). For instance, influenza A is now known to have 18 subtypes of HA and 11 subtypes of NA (Li et al., 2012; Tong et al., 2012; Wu et al., 2014). This high mutation rate and the emergence of new strains can also make vaccine development and policy difficult to plan and carry out. Due to viral antigenic shift, yearly influenza vaccines are required so the population is sufficiently protected by the vaccine, however, vaccine composition is determined ∼8 months in advance of administration. This lag may allow new strains to emerge or for antigenic drift to result in a poor match between vaccine and the circulating strain of influenza. Furthermore as seen in the 2009 influenza pandemic, governments and public health departments face considerable difficulties in the production and distribution of vaccines when faced with sudden or unexpected outbreaks of newly emerged strains (Houser and Subbarao, 2015).

A common complication of respiratory viral disease can be secondary bacterial infection. Noting this association is important as it has clear implications for global health, principally because bacterial co/secondary infection is known lead to increased morbidity (Smith and McCullers, 2014). Co/secondary bacterial infection, as the name suggests, is a bacterial infection that occurs during or after an infection from another pathogen, commonly viruses. A number of viral infections (including infection from influenza virus, respiratory syncytial virus, parainfluenza virus and human metapneumovirus) can be complicated by co/secondary infection by a variety of bacteria including *Streptococcus pneumoniae, Haemophilus influenzae*, and *Staphylococcus aureus*. This association leads to an increased severity of disease and sequela such as pneumonia (Smith and McCullers, 2014). In this review we dwell on influenza pandemics since the late 1800’s, focussing on the associations and complications that arise from secondary bacterial infections.

## Influenza

Influenza viruses are important zoonotic pathogens as they are highly contagious and one of the most prevalent causes of respiratory infection. Worldwide annual epidemics reportedly cause up to five million cases of severe illness, which result in 250,000–500,000 deaths per year. The majority of deaths caused by influenza occur in young children and people over 65 (World Health Organization, 2016). Reports suggest that each year up to 20% of the United States population may be infected by influenza (Sullivan et al., 1993; Biggerstaff et al., 2014). The virus spreads easily from person to person via aerosol droplets (Hilleman, 2002; Taubenberger and Morens, 2008) and replicates in the upper and lower respiratory tract (Taubenberger and Morens, 2008). Commonly, in non-tropical regions, annual influenza epidemics occur during late autumn and winter. Although less frequent, tropical regions too suffer influenza epidemics, these generally coinciding with the rainy season (Cox and Subbarao, 2000; Biggerstaff et al., 2014).

There are three types of influenza virus, types A, B, and C, each differing in host range and pathogenicity (Taubenberger and Morens, 2008). Type A has been isolated from humans, avian, swine, horses, mink, dogs, seals, and ferrets (Jakeman et al., 1994; Taubenberger and Morens, 2008; Parrish et al., 2015), whilst type B has been isolated from humans, seals (Osterhaus et al., 2000) and ferrets (Jakeman et al., 1994), and type C from humans (Matsuzaki et al., 2002), swine and dogs (Youzbashi et al., 1996). Influenza A and B virions contain several structural antigens and three antigenic surface proteins; HA, NA, and M2/BM2 ion channels (Webster et al., 1992; Hampson and Mackenzie, 2006; Racaniello, 2009; Dormitzer et al., 2011). Influenza virus C only expresses one antigenic surface protein, haemagglutinin-esterase-fusion (HEF), and thus stimulates a lesser immune reaction than types A or B (Taubenberger and Morens, 2008; Racaniello, 2009). Influenza A is the fastest to evolve, at a rate 2–3 times faster than B, whilst C is the slowest (Yamashita et al., 1988). Antigenic drift allows the influenza virus to escape immunity acquired through previous exposure or vaccination; thus influenza A causes more epidemics and pandemics than either influenza B or C (Hampson and Mackenzie, 2006; Taubenberger and Morens, 2008). Whilst influenza B causes periodic/yearly epidemics but not pandemics, influenza C viruses only cause relatively infrequent mild respiratory problems (Taubenberger and Morens, 2008). Throughout the past 300 years there have been 12 pandemics caused by influenza A; the most infamous being the 1918 ‘Spanish flu’ pandemic (Taubenberger and Morens, 2008). In the years between 1933 and 1957 there were nine influenza A (H1N1) epidemics and five influenza B epidemics. The worst of all these epidemics was the 1935–1936 influenza B epidemic that resulted in at least 55,000 deaths. This was closely followed by the 1943–1944 influenza A (H1N1) epidemic which caused 53,000 deaths (Glezen, 1996). Evidently, although influenza B doesn’t cause pandemics, it is still a cause for concern.

During an infection influenza virions attach to and enter host epithelial cells by the binding of viral HA to sialic acid on the host cell which instigates endocytosis and the movement of the virion into the cell within an endosome. The virus then uses/hijacks the host cells ‘machinery’ to replicate and transcribe viral RNA and produce more viral components (Samji, 2009). Progeny virions bud from the host cell, using the host cell membrane as a viral envelope, and go on to infect neighboring host cells (Nayak et al., 2009). As influenza infection develops the virus causes cell damage and death within the host’s airways and up-regulates the production of toxins, causing further destruction. Influenza cytotoxins for example causes necrosis of host cells (Conenello and Palese, 2007; Iverson et al., 2011). Influenza infection, particularly pandemic influenza infection, is known to generate an increased inflammation response within the host, as the body works to rapidly deliver immune cells to the site of infection. This inflammation is a response to the expression of cytokines and chemokines (de Jong et al., 2006; Kash et al., 2006; Kobasa et al., 2007; Rock and Kono, 2008). Virally induced decreased mucociliary activity, the dysfunction of immune cells and the reduction of phagocytosis reduces clearance of the virus from the host airways and the host’s ability to fight the virus (Brundage, 2006; Wu et al., 2011; Cauley and Vella, 2015). In an attempt to limit and control infection, the host immune system kills infected host cells. It does this in several ways, including; the production of perforin by Natural Killer (NK) cells which creates lesions/pores in cell membranes resulting in the induction of apoptosis, apoptosis from tumour necrosis factor (TNF) and FasL and the production of reactive oxygen species from macrophages and neutrophils causing oxidation of cellular lipids, proteins and DNA resulting in cell dysfunction and death (Topham and Hewitt, 2009; Kash et al., 2014; Kash and Taubenberger, 2015). Of course viral infection and/or interference with host processes can cause and direct the pathway of cell death, as is the case for necrosis. Host cell death, whether apoptosis, necrosis or pyroptosis, impacts on the severity and outcome of influenza disease in a variety of ways. Virally induced death of immune cells assist in the evasion of host defenses and hinders the clearance of the virus promoting the development of infection. Studies have shown a 90% reduction of alveolar macrophages in mice within a week of influenza infection, and evidence of necrosis in the remaining macrophages (Robinson et al., 2015). Necrosis and pyroptosis are pro-inflammatory due to their role in the release of cytokines. These cell death pathways allow for the rapid release of intracellular contents, including any viral components, from the infected host cell promoting host inflammatory responses and the formation of a cytokine storm which causes host tissue damage (Cundell et al., 1995; Rock and Kono, 2008; Lamkanfi and Dixit, 2010; Cauley and Vella, 2015). Furthermore infection with some influenza subtypes, for instance H1N1 and H5N1, typically result in lymphopenia, a state of abnormally low levels of lymphocytes, which has been associated with higher viral load. de Jong et al. (2006) found influenza infection caused lower levels of cytotoxic T cell lymphocytes, which would therefore negatively affect acquired immunity (de Jong et al., 2006; Cunha et al., 2009). Where lymphopenia occurs, studies have shown a corresponding increase in macrophages. Supporting the evidence for the increase in macrophages is the significant increase in IP-10 (a chemokine secreted in response to gamma interferon (IFNγ) which activates macrophages), Interleukin-8 (IL-8, a chemokine which is produced by macrophages), IL-6 (in this case, a pro-inflammatory cytokine secreted by macrophages), and MCP-1 (a chemokine that recruits monocytes, a type of leukocyte that can differentiate into macrophages) (de Jong et al., 2006; Kobasa et al., 2007).

## Influenza Pandemics Since the Late 1800’s

**FIGURE 1:**
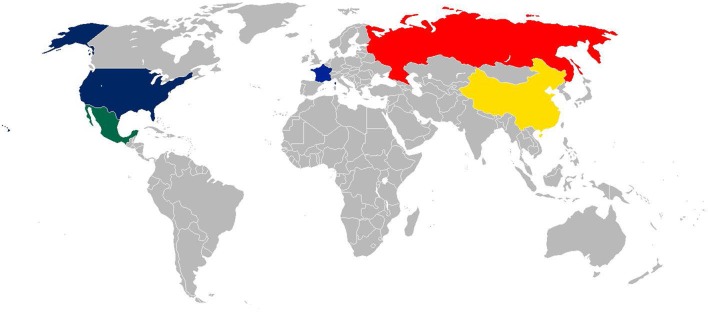
World Map showing countries confirmed and suspected of being the origin of influenza pandemics. Blue – The origin of the 1918 Spanish is still unclear, although various papers suggest the United States (New York) or France as the origin; yellow – China the origin of the 1957 Asian flu pandemic; Hong Kong, the origin of the 1968 Hong Kong pandemic; red – Russia, the origin of the 1889 and 1977 Russian flu pandemics; green – Mexico, the origin of the 2009 Swine flu pandemic.

Influenza pandemics, generally characterized by the emergence of a novel influenza A against which little or no immunity exists within the global populace, are a cause of high mortality and morbidity and are a major financial burden (Glezen, 1996). Since the 1800’s these pandemics have arisen from a number of countries, spreading across the globe (**Figure 1**). Detailed below and in **Table 1** we have sought to describe some of the most significant influenza pandemics since the late 1800’s to highlight the potential impact of influenza with respect to associations with bacterial infection.

**Table 1:** Details of significant influenza pandemics since the late 1800’s.

Name of pandemic	Year	Strain	Disease burden	Additional information
Russian influenza pandemic	1889	A(H2N2)	132,000 deaths in England, Wales, and Ireland alone (Smith, 1995).	The 1889 ‘Russian Flu’ as the name suggests started in Russia and spread across Europe reaching North America in 1890. In only 4 months the infection had spread across all of Europe and the United States. The United Kingdom encountered four waves of disease and it is thought that at least one third of the adult population in England and Ireland suffered at least one bout of disease (Smith, 1995; Valleron et al., 2010).
Spanish influenza pandemic	1918	A(H1N1)	Caused 40–50 million deaths worldwide. India alone suffered 7 million deaths (Potter, 2001; Hilleman, 2002; Brundage, 2006; Michaelis et al., 2009).	Considered the most devastating influenza pandemic ever recorded, infecting 50% of the world’s population. The origin of this pandemic is unclear as it appeared in North America, Asia, and Europe at roughly the same time (Taubenberger et al., 2001; Hilleman, 2002). Reports of disease and mortality were initially suppressed in many countries, included the United Kingdom, France and the United States, to ensure wartime efforts and morale weren’t negatively affected. In Spain the press were able to print freely, meaning the first publicized cases were reported from Spain facilitating the nickname ‘the Spanish flu’ (Johnson, 2016; Peckham, 2016). In contrast to its name, it has been suggested that the pandemic started in France/mainland Europe and that it reached Spain from France (Reid et al., 2001; Trilla et al., 2008) although more recent papers suggest New York as the origin due to evidence of a pre-pandemic wave of the H1N1 virus (Olson et al., 2005). What is remarkable is how far the pandemic spread; the pandemic reached as far as the Alaskan wilderness to remote Pacific islands (Burnet and Clark, 1942; Taubenberger et al., 2001). The pandemic experienced a couple of waves; the first of which was relatively mild. The second wave, however, was far more lethal (Hilleman, 2002). The first outbreaks were reported in military camps as males responded to the call for troops in the spring and summer of 1918. A period of dormancy was then recorded toward the end of summer in America, but this was short lived as transmission picked up as schools reopened in September after the summer holidays (Glezen, 1996).
Asian influenza pandemic	1957–1958	A(H2N2)	Although global death toll estimates vary [between 1.5 million (Gatherer, 2009) and 2–4 million (Michaelis et al., 2009)], the death toll in the United States is accurately reported to have been 69, 800 (Klimov et al., 1999; Hilleman, 2002).	The pandemic affected 40–50% of people worldwide (Potter, 2001), however, resulted in lot less mortality than the previous pandemic. This Asian influenza pandemic started in March 1957 in Southern China, where pigs, ducks, and humans live together closely. It reached Hong Kong in April, and then spread to Singapore, Taiwan, and Japan (Fukumi, 1959; Potter, 2001; Hilleman, 2002). The pandemic reached India, Australia, and Indonesia by May, Pakistan, Europe, North America, and the Middle East by June, South Africa, South America, New Zealand, and the Pacific Islands by July, and Central, West and East Africa, Eastern Europe, and the Caribbean by August (Dunn, 1958; Payne, 1958; Potter, 2001).
Hong Kong influenza pandemic	1968–1969	A(H3N2)	1–2 million people died worldwide (Michaelis et al., 2009). Overall 33,800 people died in the United States (Klimov et al., 1999) and England and Wales saw a 55% increase in respiratory deaths in 1969 (Tillett et al., 1983).	The 1968 Hong Kong pandemic started in July 1968 in Hong Kong and spread to the Southern hemisphere by June 1969 (Biggerstaff et al., 2014). The H3N2 virus was isolated and identified too late in the pandemic for vaccine intervention (Nakajima et al., 1978; Hilleman, 2002) so it was fortunate that in most countries, apart from the United States, the disease was mild (Cockburn et al., 1969). There are several proposed reasons for the reduced mortality of this compared to the Asian Flu. Firstly the N2 was seen in the Asian Flu so may have contributed some cross-reactive immunity to this H3N2 strain (Glezen, 1996). Although antibodies to NA do not prevent infection, they help to reduce the amount of newly formed virus released from infected cells (Couch et al., 1974; Glezen, 1996). Secondly, during the initial wave of this pandemic, the number of cases started to grow exponentially in December, at this point the school Christmas holidays began; it has been speculated that this removed an important susceptible population (Glezen, 1996).
Russian Flu influenza pandemic	1977–1978	A(H1N1)	Approximately 700,000 deaths globally (Michaelis et al., 2009).	This pandemic was caused by a reappearance of H1N1, identical to that of the Spanish flu virus (Michaelis et al., 2009). The disease mainly affected those born after the late 1950’s, so those who had not been exposed to the pandemic H1N1 strain that had circulated previously (Hilleman, 2002).
Swine influenza pandemic	2009	A(H1N1)	By the end of the pandemic it is thought that there were 284,000 deaths worldwide (Chertow and Memoli, 2013).	In early 2009, an influenza A H1N1 virus outbreak was initially identified in Mexico and then the United States (Michaelis et al., 2009). In June 2009 the WHO declared the outbreak a pandemic. Within 4 weeks the outbreak had spread to 41 countries, resulting in 11,034 confirmed cases and 85 deaths (Michaelis et al., 2009; Wang and Palese, 2009). Disease/symptoms were generally mild (Peiris et al., 2009) however, complications of the disease did result in hospitalization, particularly in at risk groups (Wang and Palese, 2009).
				Unlike other pandemics and yearly epidemics, during this pandemic it was predominantly children and young adults that were affected, particularly those aged 12–22 (Gill et al., 2010). Overall this pandemic was relatively mild. It is thought that morbidity and mortality rates were reduced due to three main factors. Firstly, the quick responses of various governments in terms of school closures helped reduce the spread of the virus. Thousands of schools were shut worldwide, including the United States and Mexico. Japan alone closed almost 2000 schools (Wang and Palese, 2009; Jackson et al., 2014). Secondly, influenza A H1N1 strains have been circulating amongst the human population for decades, therefore prior exposure could have provided some degree of immunity against the 2009 pandemic strain. Lastly an important pathogenicity factor, PB1-F2, was not present making the strain milder than those present in previous pandemics (Wang and Palese, 2009).


## Bacterial Co-Infection and Secondary Infections

Laboratory, clinical and epidemiological research has made it abundantly clear that bacterial co/secondary infection can significantly increase the morbidity and mortality of viral infections (Gupta et al., 2008). Up to 75% of those infected with influenza that go on to acquire pneumonia, are confirmed to have bacterial co-infection (Zambon, 2001). Bacterial co/secondary infection of influenza infection appears to occur frequently. Studies have shown that up to 65% of laboratory confirmed cases of influenza infection exhibited bacterial co/secondary infection, although Klein et al. (2016) state that in the majority of the research included in their meta-analysis this figure ranged between 11 and 35%. In the setting of an influenza epidemic or pandemic bacterial co/secondary infection can have devastating consequences, particularly in at-risk groups such as the immunocompromised/immunosuppressed. Immunosuppression is associated with more severe morbidity and a much higher risk of mortality from co/secondary bacterial infection (Rice et al., 2012). During the 2009 Swine influenza pandemic, there was an increase in hospital pneumonia cases as a result of secondary bacterial pneumonia, which was identified in 29–55% of mortalities (Centers for Disease Control and Prevention, 2009; Gill et al., 2010; Weinberger et al., 2012).

## Pathobionts Associated with CO/Secondary Bacterial Infection

The upper respiratory tract has been shown to host a diverse microbiota, within which a number of bacterial pathobionts may be found, i.e., those bacterial species that can be pathogenic yet also harmlessly carried (Hooper et al., 2012; Cauley and Vella, 2015). *Legionella pneumophila* (Rizzo et al., 2010), *Streptococcus pyogenes* (Chertow and Memoli, 2013), *Neisseria meningitidis*, *Moraxella catarrhalis*, *S. pneumoniae*, *H. influenzae*, *S. aureus* (Dela Cruz and Wunderink, 2017), *Pseudomonas aeruginosa* as well as a number of other *Streptococcus* and *Staphylococcus* spp. (Yang et al., 2016a) have all been associated with co-infection of influenza. However, *S. pneumoniae*, *H. influenzae*, and *S. aureus* are the most commonly reported bacteria associated with co/secondary infections during influenza pandemics since the late 1800’s.

### Streptococcus pneumoniae

*Streptococcus pneumoniae* is the most common bacteria found in viral secondary bacterial infections, and is particularly associated with causing high mortality and morbidity during influenza epidemics and pandemics (Brundage, 2006; Joseph et al., 2013). *S. pneumoniae* is a Gram-positive diplococci and is the most common cause of community-acquired pneumonia and invasive disease, i.e., sepsis and meningitis worldwide, as well as less severe acute disease such as otitis media (Bridy-Pappas et al., 2005; McCullers et al., 2010). *S. pneumoniae* is grouped into >97 immunologically distinctive serotypes based on a polysaccharide capsule (Bentley et al., 2006; Park et al., 2007; Jin et al., 2009; Calix and Nahm, 2010; Calix et al., 2012). A burden to public health in its’ own right, the WHO has reported that diseases caused by *S. pneumoniae* resulted in approximately 826,000 deaths in 2000 alone (Pittet and Posfay-Barbe, 2012). A more recent study shows that there are 4 million cases of disease caused by *S. pneumoniae* and 22,000 deaths annually in the United States (Huang et al., 2011). The current public health impact of *S. pneumoniae* infection is reduced by vaccine policies, with, for example, PCV-13 and PPV-23 being used for children and adults, respectively, in the United Kingdom (Pittet and Posfay-Barbe, 2012).

Many studies have shown that influenza infection facilitates the acquisition, colonization and development of disease from *S. pneumoniae* in people of all ages (Shrestha et al., 2013; Grijalva et al., 2014; Siegel et al., 2014). This is partly due to *S. pneumoniae’s* ability to catabolise sialic acid which is released from host cells and mucus by influenza’s NA. Influenza infection also results in increased mucus production, further increasing the amount of metabolite available for *S. pneumoniae*. The NA produced by *S. pneumoniae* also assists in the release of sialic acid (Siegel et al., 2014). Mouse models support the concept that influenza facilitates the development of disease from *S. pneumoniae*; they have provided evidence that influenza infection enhances secondary *S. pneumoniae* pneumonia (McCullers and Rehg, 2002; McCullers and Bartmess, 2003). Wu et al. (2011), showed that co-infection of a virus and a bacterium can either occur from mixed viral bacterial infection, or from a viral infection being sequentially followed by a bacterial infection. Sequential bacterial infection normally occurs within a 7-day period of the viral infection. Influenza infections and successive *S. pneumoniae* infections result is a time and dose dependent change in the host dendritic cells which produces enhanced inflammation. Berendt et al. (1975) inoculated squirrel monkeys with either influenza A, *S. pneumoniae* or influenza A and *S. pneumoniae*. Influenza alone caused minor illness such as mild tracheitis, with symptoms such as sneezing, coughing and fever (although some did develop bronchopneumonia) and had a 100% survival rate. *S. pneumoniae* again caused minor illness with a 100% survival rate. Co-infection of influenza A with *S. pneumoniae* resulted in severe morbidity with a 75% death rate within 40 h, clear evidence of the consequences of co/secondary bacterial infection (Berendt et al., 1975). These findings are reflected in several other studies, with some even showing that co-infection may assist in the spreading of *S. pneumoniae* infection to the lower respiratory tract (Takase et al., 1999; Seki et al., 2004).

An additional mouse model of infection provided comparable results whilst comparing the effect of different *S. pneumoniae* serotypes on co-infection (Sharma-Chawla et al., 2016). More cases of pneumonia and bacteraemia were observed in mice infected with both influenza A and *S. pneumoniae* than in mice infected with these pathogens individually. This was the case for all *S. pneumoniae* serotypes tested. More virulent pneumococcal serotypes caused a greater burden of disease in both the co-infected mice and those infected with *S. pneumoniae* alone. The highly invasive pneumococcal serotype 4 caused pneumonia in 58% of mice and bacteraemia in 21% in a single infection model. When co-infecting with influenza these figures increased to 100 and 90% for pneumonia and bacteraemia, respectively. Mortality rates increased from 0% for individual infection to 79% during co-infection. In comparison, individual infection with a carrier strain (of lower invasive potential) of serotype 19F, caused pneumonia in 91% of cases and bacteraemia in 0%. When co-infecting with influenza and 19F these figures increased to 100 and 33%. Mortality rose from 0% during individual infection to 63% during co-infection (Sharma-Chawla et al., 2016).

Pneumococcal vaccination has shown to ameliorate the risk of secondary bacterial pneumonia. During a vaccine efficacy study, the incidence of pneumonia in those with influenza reduced by 45% in groups vaccinated against *S. pneumoniae* (Madhi et al., 2007). However, whilst vaccine implementation has successfully reduced pneumococcal disease in a number of countries, lower levels of vaccine implementation in low and middle income countries coupled with fractional serotype coverage and increasing levels of antibiotic resistance, means the specter of influenza pandemic associated *S. pneumoniae* secondary infection remains a significant risk to global health.

### Haemophilus influenzae

*Haemophilus influenzae* is another bacteria commonly found to co/secondarily infect viral infection, and has been associated with the complication of disease during influenza pandemics (Abrahams et al., 1919; Spooner et al., 1919; Brundage, 2006; Palacios et al., 2009). It is a Gram-negative fastidious coccobacillus. Typeable strains have a polysaccharide capsule and are categorized into six serotypes (A–F). *H. influenzae* serotype B was a major cause of invasive disease (Wenger, 1998; Murphy, 2003; Chin et al., 2005; Brouwer et al., 2010) although widespread implementation of the Hib vaccine has significantly reduced the burden of disease (Rosenstein and Perkins, 2000). Those *H. influenzae* that lack a capsule, denoted non-typeable *H. influenzae* (NTHi), remain a significant cause of bacterial meningitis, otitis media and exacerbations of chronic lung disease such as COPD worldwide (Langereis and de Jonge, 2015).

Various studies have shown the impact when *H. influenzae* co/secondarily infects with influenza, and some suggest a level of synergism. The effect of influenza and *H. influenzae* co-infection verses individual infection of both pathogens is tellingly different; Shope found that co-infection resulted in severe disease or death when on their own *H. influenzae* and influenza only caused mild infection or disease (Shope, 1931). More recently, Lee E.H. et al. (2010) undertook a similar study which provided comparable results and evidence that influenza and *H. influenzae* co-infection produces more epithelial cell destruction than single infection with either pathogen (Lee L.N. et al., 2010). Furthermore, they found individual infection caused mild bronchiolitis within 4 days of initial infection, from which the host lung was able to recover. Conversely, co-infection caused bronchial necrosis, bronchial inflammation and bronchitis within the same time period or less, and led to further complication such as epithelial erosion (Lee L.N. et al., 2010). It is now commonly accepted that co-infection results in more severe morbidity and poorer clinical outcome than infection of influenza *or H. influenzae* alone.

Further support of the impact of co-infection comes from Michaels et al. (1977), who dosed two groups of rats intranasally with *H. influenzae* with the intention of giving them meningitis. One group of rats were naive and the other had previously been dosed with influenza. In both groups ∼50% of the rats acquired meningitis, however, the naïve rats required a 100-fold larger dose of *H. influenzae* (Michaels et al., 1977).

As is the case for many bacterial and viral co-infections, mortality from *H. influenzae* and influenza co-infection is highly dependent on the timing of the introduction of the secondary microbe as well as density of bacterial colonization. Studies have shown that when influenza virus and *H. influenzae* are introduced at the same time there is no synergistic relationship. When *H. influenzae* is introduced 7 or more days after influenza there is again no synergistic relationship; however, high lethality is exhibited when *H. influenzae* and influenza are introduced 3 or 4 days apart (Lee L.N. et al., 2010).

### Staphylococcus aureus

*Staphylococcus aureus* is a Gram-positive cocci that has been found to complicate influenza infection; increasingly so in more recent years/pandemics (Hers et al., 1958; Palacios et al., 2009). *S. aureus* is transiently carried in the nose of 30% of the population, whilst 20% of the population have persistent nasal colonization (Wertheim et al., 2005). Like *H. influenzae* and *S. pneumoniae*, *S. aureus* is an opportunistic pathogen and a major cause of bacteraemia (Wertheim et al., 2005; Tong et al., 2015). It is also a common cause of pneumonia (Kollef et al., 2005); specifically necrotising pneumonia that is caused by community acquired Methicillin-resistant *Staphylococcus aureus* (MRSA) and has a 30% mortality rate (Murray et al., 2010). Necrotising pneumonia is highly associated with either the presence of Panton-Valentine leukocidin (PVL) or prior/co influenza infection (DeLeo and Musser, 2010). MRSA is a particularly problematic pathogen and concern for public health as it can be hard to treat due to its multidrug-resistant properties (Wu et al., 2005; Eom et al., 2014; Fishovitz et al., 2014).

Influenza infection has been shown to increase the adherence of *S. aureus* (as well as *H. influenzae* and *S. pneumoniae*) to host pharyngeal cells (Fainstein et al., 1980). In addition to this, mouse models have highlighted increased morbidity and mortality in mice that are pre-infected with influenza before they are exposed to *S. aureus* vs. those just exposed to *S. aureus*. Increased lung damage and bacterial density has also been shown (DeLeo and Musser, 2010; Lee M.H. et al., 2010; Iverson et al., 2011). Lee M.H. et al. (2010) showed that mice infected with low doses of influenza, low doses of *S. aureus* and high doses of *S. aureus* were able to survive. Those infected with high doses of influenza died within 4–7 days; however, all mice infected with a high dose of influenza and then a high dose *S. aureus* died within 2 days of bacterial exposure, showing how death can be accelerated by co-infection. When mice were infected with a low dose of influenza and then a high dose *S. aureus* they died at 7 days. The fact that the mice survived low influenza infection on its own, but could not survive co-infection with *S. aureus* shows the lethality of such co/secondary bacterial infection (Lee M.H. et al., 2010).

In an act of synergism, *S. aureus* infection may actually assist influenza infection by increasing the infectivity of influenza; when the virion is being moved into the host cell within an endosome the low pH in the endosome causes a conformational change to the HA [HA_(0)_] allowing it to be cleaved by host proteases into two subunits [HA_(1)_ and HA_(2)_]. This cleaving ‘activates’ the HA, mediating fusion between the virus and endosome membrane, ready for the opening of the M2 ion channel so the vRNP (viral ribonucleoproteins) can be released into the host cell where the viral RNA is replicated and transcribed. Several strains of *S. aureus* produce proteases that cleaves influenza HA; the more protease that is available, the more HA can be cleaved meaning more vRNP can get into host cells meaning overall more progeny virions (Tashiro et al., 1987; Steinhauer, 1999; Samji, 2009). This aspect contributes to the increased severity of disease caused by co-infection verses individual influenza infection. And although not all strains of *S. aureus* produce proteases that cleave influenza HA, the proteases they do produce indirectly enhance morbidity by causing host inflammatory responses which result in the production of host enzymes that are capable of cleaving HA (Tashiro et al., 1987).

## Historical Evidence of CO/Secondary Bacterial Infection During Major Influenza Pandemics

### 1918 Spanish Influenza Pandemic

The 1918 influenza pandemic was a result of influenza strain A (H1N1). It is considered the most devastating influenza pandemic ever recorded, infecting 50% of the world’s population and resulting in approximately 40–50 million deaths worldwide. India alone suffered 7 million deaths (Potter, 2001; Hilleman, 2002; Brundage, 2006; Michaelis et al., 2009). The main groups of individuals affected by this pandemic were those aged 20–40 years old, in addition to infants and those over 65. Ordinarily only young children and the elderly are the age groups most at risk from influenza, showing how distinctive pandemic strains can be (Potter, 2001). It is suggested that war time efforts meant that influenza easily spread through military camps, allowing the 20–40 years old age range to be more at risk than usual.

There are many published examples of co/secondary bacterial infections during the 1918 influenza pandemic, and pneumonia as a consequence of bacterial infection is estimated to have occurred in up to 95% of deaths during this pandemic (Morens et al., 2008). A majority of those deaths due to secondary *S. pneumoniae* infection (Brundage and Shanks, 2008; Morens et al., 2008). Many of the examples that detail co/secondary bacterial infection come from outbreaks within army camps. Within a 1-month period in 1918 at the military Camp Devens, a quarter of all troops were diagnosed with influenza. Of those infected, 17% developed pneumonia, of which 35% of cases were fatal. Out of 37 autopsies performed, 43% were positive for pure growth of *H. influenzae* in at least one lobe of the lung. Blood culture revealed 65% had *S. pneumoniae*, 2.5% had *H. influenzae* and 1.3% had *S. aureus* (Spooner et al., 1919; Brundage, 2006). This pattern of invasive bacterial co/secondary infection has also been documented for several other camps during the same year, including Camp Logan. Here 2,487 influenza-associated hospitalizations were recorded, 17% acquired pneumonia with 4% of these cases being fatal. Post-mortems found *S. pneumoniae* in the lungs of 44% and heart blood of 33% (Hall et al., 1918; Brundage, 2006). At Camp Jackson, 17% of influenza cases progressed to pneumonia with a further 31% of pneumonia cases proving fatal. Autopsies found *S. pneumoniae* to be the bacterial co-infection most associated with pneumonia, however, 155 of 312 lung cultures were positive for *S. aureus* (Michael, 1942; Brundage, 2006). At Camp Custer, 21% of influenza cases progressed to pneumonia, of which 28% died. Sputum cultures proved the presence of *S. pneumoniae* in 26% of cases. Further investigation found 28% of lung and blood cultures were positive for *S. pneumoniae*, again acting as supporting evidence of the invasive potential of such co-infections (Blanton and Irons, 1918; Brundage, 2006). Camp Fremont experienced 2418 hospitalizations, 17% had pneumonia of which 36% were fatal. Nasopharyngeal and sputum samples from 158 pneumonia cases found *S. pneumoniae* in 41% of cases, *H. influenzae* in 38%, and other *Streptococcus* spp. in 29% (Brem et al., 1918).

Further lung tissue from fatalities of this pandemic were re-examined in 1919; *S. pyogenes longus* was found in 36% of cases, *S. pneumoniae* in 29% of cases and *H. influenzae* in 25% (Abrahams et al., 1919; Brundage, 2006). Additional post-mortems of lung tissue suggest that at least 90% of samples showed evidence of bacterial infection (Oxford et al., 2002; Morens et al., 2008; Chien et al., 2009). Overall 95% of deaths were due to co/secondary bacterial pneumonia (Opie et al., 1921; Morens et al., 2008).

Co-infection had also been reported as an issue prior to the official start of the pandemic. Influenza with secondary bacterial infection of *S. pneumoniae* (and other *Streptococcus* sp.), *H. influenzae* and/or *Staphylococcus* sp. was associated with major outbreaks of purulent bronchitis in 1916 and 1917 (Brundage, 2006; Joseph et al., 2013). Indeed in 1916–1917 British, Australian, Canadian, and American armed forces in England and France experienced an epidemic of purulent bronchitis. Out of 20 tested sputum specimens from a British army camp based in north France, 90% presented with *H. influenzae*, 65% presented with *S. pneumoniae*, 25% with other *Streptococcus* spp. and 15% with *Staphylococcus* spp. Out of the specimens positive for *H. influenzae*, many exhibited simultaneous *H. influenzae* and *S. pneumoniae* co-infection; with *H. influenzae* identified as the primary bacterial infector. *S. pneumoniae* infection first presented with low virulence, however, pathogenesis soon worsened, it has been suggested, as result of the symbiotic growth with *H. influenzae* (Brundage, 2006; Dennis Shanks et al., 2012). Of course it is known that there is a positive association between the colonization of *H. influenzae* and *S. pneumoniae*, and colonization is a prerequisite for disease, so the presence of such co-infection fits with current knowledge (Jacoby et al., 2007; Abdullahi et al., 2008).

### 1957 Asian Influenza Pandemic

This pandemic affected 40–50% of people worldwide. The cause was influenza strain A (H2N2) (Potter, 2001). Although global death toll estimates vary [between 1.5 million (Gatherer, 2009) and 2–4 million (Michaelis et al., 2009)], the death toll in the United States is accurately reported to have been 69, 800 (Klimov et al., 1999; Hilleman, 2002). Post-mortem cultures show evidence of bacterial infection in up to 80% of all severe and fatal cases (Hers et al., 1958; Morens et al., 2008; Gill et al., 2010).

During this pandemic the United States, and many other countries, experienced an increase in hospitalization rates. A majority were due to pneumonia, predominantly caused by *S. pneumoniae*, *H. influenzae*, and *S. aureus* infection (Petersdorf et al., 1959). There are similar documented reports from the Netherlands; of the 148 deaths presumed to be from the Asian pandemic influenza strain that were examined fully, 75% presented with bacterial pneumonia of which 15% were positive for *S. pneumoniae* and 59% were positive for *S. aureus* (Hers et al., 1958).

Robertson et al. (1958) unveiled similar findings when investigating the hospitalization of 140 people suffering pneumonia at Sheffield City General Hospital in 1957. A majority showed evidence of influenza A infection; 27% of those had co/secondary infection of *S. aureus* (which had a 47% death rate), 15% *S. pneumoniae* and 4% *H. influenzae*, although this is likely to be an underestimation as many patients had already started taking antibiotics (Robertson et al., 1958).

### 1968–1969 Hong Kong Influenza Pandemic

Worldwide 1–2 million people died during this pandemic which was caused by the influenza strain A(H3N2) (Michaelis et al., 2009). Although this is a lower death toll than engendered in previous pandemics, it is still an awfully high number of deaths. Overall 33,800 people died in the United States (Klimov et al., 1999) and the pandemic cost 3.9 billion dollars (Hilleman, 2002). In 1969, England and Wales saw a 55% increase in respiratory deaths, of which co/secondary bacterial infection was shown to be a major contributor (Tillett et al., 1983).

Staphylococcal pneumonia in particular was a major source of complication to influenza infection. A hospital in Atlanta suffered a threefold increase in cases of Staphylococcal pneumonia during this pandemic. Staphylococcal infection caused 26% of pneumonia cases during this period, and a high correlation was recognized between influenza infection and bacterial pneumonia (Schwarzmann et al., 1971). In addition, out of 79 cases of fatal influenza with pneumonia complications, 16% had bacterial co-infection with *S. pneumoniae* (6%), *S. pyogenes* (5%), and *S. aureus* (1%) being the main causes. More than one of these bacteria were present in 4% of cases (Schwarzmann et al., 1971; Metersky et al., 2012).

Another health care facility in the United States, the Mayo Clinic in Minnesota, also found *S. aureus* to be a major cause of complication. Of 129 adults diagnosed with pandemic influenza, pneumonia was established in 16%, of which 40% of these cases (6% of all 129 influenza cases) were fatal. *S. aureus* or *P. aeruginosa* bacterial infection was present in 75% of all fatal cases, indicating bacterial co/secondary infection was a major determinant of severe disease and death (Lindsay et al., 1970).

In previous pandemics *S. pneumoniae* has been proposed as the major contributor of mortality and morbidity, however, during this 1968–1969 Hong Kong and the 1957 Asian influenza pandemic *S. aureus* clearly had a larger impact. This is possibly a reflection of increased antibiotic use and increased antibiotic resistance.

### 2009 Swine Influenza Pandemic

Within 4 weeks this outbreak of influenza A(H1N1) had spread to 41 countries resulting in 11,034 confirmed cases and 85 deaths (Michaelis et al., 2009; Wang and Palese, 2009). By the end of the pandemic it is thought that there were 284,000 deaths worldwide, with Mexico and the United States being most severely affected (Chertow and Memoli, 2013). Unlike other pandemics and yearly epidemics, during this pandemic it was predominantly children and young adults that were affected, particularly those aged 12–22 (Gill et al., 2010). Influenza A (H1N1) strains have been circulating amongst the human population for many years therefore this prior exposure could have provided many adults with some degree of immunity against the 2009 pandemic strain, particularly older groups who were more likely exposed during previous pandemics.

Surveillance by the New York City Department of Health and Mental Hygiene has shown that during the 2009 Swine Flu Pandemic almost 30% of the first 47 deaths showed invasive bacterial disease. *S. pneumoniae* was the most common causative agent identified (followed by *S. pyogenes*) (Lee E.H. et al., 2010). In the United Kingdom, of the 457 fatalities 68 were autopsied. Of these, 41% were shown to have complications associated with secondary bacterial infection, most commonly (25% of cases) due to *S. pneumoniae* (Lucas, 2010).

Further studies in the United States have reviewed 77 deaths during the period of May–August 2009 and found bacterial co-infection in almost 30% of cases; 46% of which were with *S. pneumoniae*, 9% with *S. aureus* and 1% with *H. influenzae* (Centers for Disease Control and Prevention, 2009). Studies based in Argentina produced similar evidence for the presence of bacterial infection, showing this wasn’t just a localized trend. Palacios et al. (2009) examined nasopharyngeal swab samples from almost 200 cases of pandemic influenza. *H. influenzae* was found in 52%, *S. pneumoniae* was found in 31% and *S. aureus* in 18% of samples. Although not the most common bacteria found, *S. pneumoniae* was the most strongly associated with severe disease (Palacios et al., 2009).

Additional research in pediatric intensive care units in the United States, investigated 838 critically ill children who were infected with pandemic influenza. Within 72 h of admission to the intensive care unit 33% exhibited bacterial co-infection; in 26% of these cases *S. aureus* was identified as the cause (48% of which were MRSA), 5.5% were positive for *S. pneumoniae* and 5% were positive for *H. influenzae.* Bacteraemia was observed in 5% of admissions, for which *S. aureus* was the main cause (Randolph et al., 2011). This study highlights how quickly co/secondary bacterial infection can become invasive particularly in at risk groups such as young children or the elderly. A point of concern is that almost half of the *S. aureus* were MRSA, and therefore inherently resistant to multiple antibiotics.

In another study of vulnerable and critically ill children in a pediatric intensive care unit in the United States, 51% of those with influenza infection had bacterial co/secondary infection. Of these 35% presented with *S. aureus*, 18% *P. aeruginosa*, 18% *M. catarrhalis*, 9% NTHI, 6% *S. pneumoniae* and 6% Group A Streptococcus. Those with *S. aureus* showed more severe morbidity and were more likely to develop disseminated intravascular coagulation which leads to a compromised blood flow within body tissue and therefore tissue damage (Nguyen et al., 2012).

In a retrospective study of 50 patients who were infected during pandemic influenza, 28% showed co/secondary bacterial infection (Dhanoa et al., 2011). *Mycoplasma pneumoniae* was found in 10%, making it the most common co/secondary infecting bacteria. This was followed by *S. aureus* found in 6%, *K. pneumoniae* and *S. pneumoniae* found in 4% and *M. catarrhalis*, *P. aeruginosa*, *S. pyogenes*, and *Streptococcus agalactiae* found in 2% of these patients (Dhanoa et al., 2011).

*Moraxella catarrhalis* is a bacteria of increasing importance being now acknowledged as the third most common cause of otitis media (OM), after *S. pneumoniae* and *H. influenzae* (Bluestone, 1986; Faden et al., 1994; Kilpi et al., 2001; Dupont et al., 2010) and the second most common cause of exacerbations in COPD, accounting for up to 4 million exacerbations per year in the United States alone (Murphy et al., 2005). *M. catarrhalis* is a cause of pneumonia (Berg and Bartley, 1987; Hager et al., 1987; Marchant, 1990; Verduin et al., 2002) and invasive disease such as bacteraemia (Ioannidis et al., 1995) and meningitis (Newing and Christie, 1947), with bacteraemia being a common complication of pneumonia, particularly in adults (Collazos et al., 1992; Ioannidis et al., 1995). Although this review has focused on *S. pneumoniae, H. influenzae*, and *S. aureus*, it has cited other bacteria seen as a source of co-infection during the various pandemics described. In early influenza pandemics such as the 1918 Spanish pandemic, *M. catarrhalis* rarely appears to be a noted cause of co-infection. However, in the 2009 pandemic it is seen in up to 18% of cases (Nguyen et al., 2012). We have therefore considered the importance of this. Data produced toward the end of the 1970’s and throughout the 1980’s demonstrated *M. catarrhalis’* potential to cause disease, however, before this *M. catarrhalis* was considered a non-pathogenic harmless commensal (McNeely et al., 1976; Johnson et al., 1981; Onofrio et al., 1981; McLeod et al., 1983; Feder and Garibaldi, 1984; Hager et al., 1987; Catlin, 1990). Therefore there are two possibilities to consider; perhaps *M. catarrhalis* wasn’t present in early pandemics as a cause of co-infection and has become more of an issue in recent years; possibly as a result of vaccines, i.e., Hib and PCV, reducing the disease burden of other bacteria such as *S. pneumonia* and *H. influenzae*. Alternatively, we must consider that as *M. catarrhalis* was not considered a pathogen it was therefore missed or not commented upon prior to the 1980’s. Retrospective studies may be able to address this. For example, autopsies from the 1918 pandemic were reviewed and it was found that *S. pneumoniae* was the most common co-infector, followed by *S hemolytic*, *S. aureus*, and *H. influenzae*. ‘Other bacteria’ were also highlighted within which *M. catarrhalis* was grouped (Morens et al., 2008).

Another point of consideration are changes of methodology. Pre-1983 laboratories would only undertake bacterial culture, however, in 2009 more sensitive methodology, i.e., PCR were available and commonly used in laboratories worldwide. The use of sensitive methods such as PCR, may have increased the likelihood of *M. catarrhalis* being detected, and as a known respiratory pathogen it would have been tested for, where as previously it may not have been. Alternatively maybe PCR detects bacteria that may have been out grown/not shown on a culture plate?

In contrast to *S. pneumoniae* and *H. influenzae* little research has been undertaken looking at influenza and *M. catarrhalis* co-infection and the dynamics and mechanisms of such infection. This is therefore an area worthy of future research. *M. catarrhalis* has been highlighted as a frequent source of co-infection for influenza since the early 1980’s (Klein et al., 2016). In the setting of a pandemic it may therefore have a major public health impact.

## Factors Affecting the Severity of Bacterial CO/Secondary Infection

As discussed above, co/secondary bacterial infection can result in a deterioration of clinical condition with more severe disease. The severity of co/secondary infection depends on multiple factors such as the strain of virus and serotype/strain of bacteria, the lag between viral infection and bacterial exposure and density of bacterial colonization (Lee L.N. et al., 2010; McCullers et al., 2010; Smith and McCullers, 2014).

### Virally Enhanced Colonization and Attachment of Bacteria

It has become clear that influenza, as well as other upper respiratory tract viral infections, leads not only to a greater risk of infection from bacterial pathobionts but also an increased likelihood that an individual may become colonized with bacteria such as *S. pneumoniae*, *H. influenzae* and *S. aureus* (Hament et al., 1999; Hilleman, 2002). Plotkowski et al. (1986) found enhanced colonization and adherence of *S. pneumoniae* to the tracheal cells of mice when they were infected with influenza (Plotkowski et al., 1986). Other studies have intranasally inoculated ferrets with influenza, finding prior viral infection increases colonization and adherence of *S. aureus* (Sanford and Ramsay, 1987). Furthermore, poor disease outcome has been linked to lost lung repair function and loss of basal epithelial cells, including alveolar epithelial cells; which is associated with increased bacterial attachment and apoptosis (Kash et al., 2011). Wadowsky et al. (1995) conducted a study in which adult subjects were inoculated with influenza and then screened for bacterial colonization. After 6 days 15% of the subjects were heavily colonized by *S. pneumoniae* (Wadowsky et al., 1995). Additionally, the effect of viral prevention methods further supports the idea of viruses predisposing a host to secondary bacterial infection (Peltola and McCullers, 2004; Lee et al., 2008). Studies have shown that influenza vaccination can reduce the occurrence of bacterial pneumonia (Fedson et al., 1993; Nichol et al., 1998).

### Viral Factors Implicated in Severity of Infection

Research shows that influenza A is the type most commonly associated with co/secondary bacterial infection and subtypes with NA2 traditionally result in more severe infection (Peltola et al., 2005). Although reported less, influenza B has also been associated with severe bacterial co/secondary infection (Finelli et al., 2008; Aebi et al., 2010). Various factors are known to impact the severity of viral infection, which in turn increases the likelihood of bacterial co/secondary infections; these include the type of HA and NA surface antigen. As mentioned previously, HA mediates virion binding to the host cell via sialic acid receptors. Binding is followed by endocytosis and the movement of the virion into the host cell within an endosome (Samji, 2009). HA binds to sialylated glycans found on the surface of human epithelial cells; traditionally seasonal influenza A virus binds to α2-6 sialylated glycans on cells in the upper respiratory tract whereas the highly pathogenic avian H5N1 strain binds to α2-3 sialylated glycans on type 2 pneumocytes lining lung alveoli (Shinya et al., 2006). Clearly the type of HA impacts on the site and development of infection. The low pH in the endosome causes a conformational change to the HA allowing it to be cleaved, an important step in penetrating into the host cell. Therefore HA and the availability of appropriate host proteases are determinants of infectivity (Steinhauer, 1999; Samji, 2009). Interestingly non-pathogenic and mammalian influenza HA undergoes cleavage outside of the host cell where as highly pathogenic strains are cleaved inside host cells (Steinhauer, 1999). Another example of how the type of HA can make a difference to infection, and therefore the impact of an epi- or pandemic, is that traditionally trypsin-like protease cleaves influenza HA; however, some HA types (i.e., types 5 and 7) have the ability to acquire insertional mutations at the cleavage site which changes their recognition site in such a way that specificity is broadened so more proteases are recognized (Kash and Taubenberger, 2015).

Neuraminidase enables the release of newly formed progeny virions; by hydrolysing the sialic acid and detaching it from the HA the virion becomes liberated from the host cell (Zambon, 2001). To be truly effective the NA must be complementary and share the same receptor specificity as HA, so if the viral HA binds to α2-3 sialic acid then the NA should hydrolyse α2-3 sialic acid (Baum and Paulson, 1991).

The production of viral toxins that impact host cell integrity is another important factor in the development of co/secondary bacterial infection. Influenza A virus can produce a viral cytotoxin PB1-F2 (Conenello and Palese, 2007; Iverson et al., 2011) which plays a role in increasing inflammation and therefore host cell damage and bacterial adherence, increasing mortality and morbidity (Lee et al., 2016). It also helps reduce bacterial clearance, increasing the occurrence and severity of co/secondary bacterial infection, by causing cell death in host monocytes (Conenello and Palese, 2007; Iverson et al., 2011).

### Molecular Co-pathogenesis

Following bacterial colonization, disease develops due to specific characteristics of viral infection that facilitate bacterial adhesion and penetration (Selinger et al., 1981). Influenza produces NA, which increases adhesion of some bacterial species by removing sialic acid to expose host cell receptors (McCullers and Bartmess, 2003; Peltola and McCullers, 2004). Alternatively some bacteria, i.e., group B Streptococci, contain sialic acid which allows for direct binding to the viral HA expressed by influenza infected host cells (Okamoto et al., 2003; Peltola and McCullers, 2004). Damaged host cells, whether damaged directly by the virus or by inflammation and immune cell responses, provide additional adhesion sites allowing for increased bacterial adhesion. For example the exposure of apical receptors like integrins permit the adhesion of bacteria such as *S. aureus* and *P. aeruginosa* (Sanford et al., 1978; Davison and Sanford, 1981; Bucior et al., 2012; Smith and McCullers, 2014). In response to viral infection, host inflammatory responses may cause an up-regulation in the expression of host receptor molecules and other molecules that bacteria can use as a receptors (Hakansson et al., 1994; Peltola and McCullers, 2004). For example Cundell et al. (1995) showed an increased presentation of G-protein-coupled platelet-activating factor (PAF) receptor, which certain bacteria, i.e., *S. pneumoniae*, can utilize for cell attachment and colonization in endothelial cells (Cundell et al., 1995; van der Sluijs et al., 2010). In contrast it has been suggested that the PAF receptor does not affect initial bacterial adherence and colonization but is more involved with assisting bacterial transition/spread into the blood and thus the development of invasive disease (McCullers et al., 2008).

Influenza infection appears to prime the host airways for bacterial infection, whilst modifying and impairing immune responses in a number of ways (Joseph et al., 2013). Viral induced immunosuppression can allow for a bacterial super infection, as host immune responses can be suppressed when immunologic cells are impaired during influenza infection and immune cell dysfunction can reduce the host’s ability to fight bacteria (Peltola and McCullers, 2004; Brundage, 2006; Wu et al., 2011). Many studies involving animal models have shown that influenza infection increases and prolongs bacterial growth, due to reduced macrophage accumulation and decreased bacterial clearance due to reduced phagocytic activity (Kleinerman et al., 1976; Wyde et al., 1989; Sun and Metzger, 2008). Additionally it has recently been shown that *S. pneumoniae* and influenza co-infection results in a reduction in the number of local alveolar macrophages, this due to increased death of these macrophages by apoptosis and necrosis (Sharma-Chawla et al., 2016). This reduction is likely to hinder bacterial clearance, hence the increased bacterial load found during co-infection during this study, and results in prolonged inflammatory response increasing morbidity. Even after influenza is cleared, *S. pneumoniae* bacterial clearance is affected. This study has highlighted some serotype dependent differences, suggesting different treatment programs would be beneficial for different serotypes. Evidence that it is worth further looking into co-infection of influenza with different serotypes of *S. pneumoniae* and other bacteria of interest (Sharma-Chawla et al., 2016). Impaired neutrophils have been shown to correlate with secondary bacterial infection in Chinchillas, due to the importance of phagocytosis during innate immunity (Abramson et al., 1981). Influenza infection is known to result in the production of IFN; pulmonary IFNγ pro-inflammatory cytokines are produced by natural killer (NK) cells as part of innate immunity and by CD4 and CD8 NK T cells as part of adaptive immunity (Schoenborn and Wilson, 2007). They increase macrophage activation during innate immunity (Scott et al., 2004) however, during T cell responses to viral infection they have been shown to inhibit bacterial clearance from the respiratory system by macrophages. It is thought that as they assist in the induction of specific anti-influenza adaptive immunity they down regulate innate immunity. The resulting suppression of phagocytosis paves the way for successful bacterial infection (Sun and Metzger, 2008). Additionally, Type I IFNs inhibit interleukin 23 (IL-23) dependent induction of T helper cell 17 (Th17) immunity, and therefore there is a reduction in the levels of CD4+ T cells and gamma delta T cells and hence a reduction in the production of IL-17 and IL-22, preventing the clearance of bacteria (Shahangian et al., 2009; Kudva et al., 2011; Mulcahy and McLoughlin, 2016). Robinson et al. (2013) have also shown that influenza A infection significantly decreased IL-1β production; IL-1β has been shown to play a role in Th17 polarization, therefore further hindering this pathway of immunity (Robinson et al., 2013). During co/secondary *S. pneumoniae* infection, type I IFNs have been shown to inhibit the production of specific chemokines (KC/CXCL1 and Mip2/CXCL2) resulting in an attenuated neutrophil response (Shahangian et al., 2009). Viral and bacterial co-infection of monocyte derived macrophages synergistically induces a pro-inflammatory response related to the type-I IFN and JAK-STAT signaling pathways (Hoffmann et al., 2016). Inflammation causes tissue damage, revealing more attachment sites for increased/developed bacterial infection. Co-infection also results in a synergistic increase in type II IFN (IFNγ) when compared to individual infection of influenza or *S. pneumoniae*, CXCL10 (aka IFNγ-induced protein 10/IP-10) is secreted in response. IP-10 attracts various immune cells including activated T cells, monocytes and macrophages, therefore causing inflammation (Dufour et al., 2002; Hoffmann et al., 2016). Patients suffering severe pneumonia show significantly higher levels of IP-10 than those with minor cases of pneumonia. IP-10 is increased during *H. influenzae* and *S. aureus* co-infection as well, and like with *S. pneumoniae*, correlates with/highlights pneumonia etiology (Hoffmann et al., 2016). In addition, when *S. pneumoniae* successively co-infects with influenza it leads to severe clinical complications; partly due to an increase in apoptosis of dendritic cells, which therefore reduces T cell priming impairing the development of adaptive immunity. Influenza and *S. pneumoniae* infections can also lead to synergistic and non-synergistic dysregulation of cytokine responses (Wu et al., 2011).

As previously described influenza infection results in a reduction in the production of IL-17 and 22. IL-17 is important in the clearance of *S. aureus* by neutrophils (Archer et al., 2013). IL-22 in involved in controlling the production of antimicrobial peptides as well as staphylococcal ligand expression (Robinson et al., 2014; Mulcahy et al., 2016). In addition to this, influenza positively affects the colonization of *S. aureus* by causing increased type III- IFN expression, which alters the IL-22 responses impairing host expression of antimicrobial peptides (Mulcahy and McLoughlin, 2016). Distress signals, such as ATP and norepinephrine, produced by damaged influenza also have several effects on *S. aureus*; namely the instigation of biofilm dispersal which helps the spread of *S. aureus* into the lungs, assisting in the development of pneumonia and invasive disease (Mulcahy and McLoughlin, 2016).

Of course viral infection doesn’t just benefit bacteria; several mechanisms of synergism between viruses and bacteria have been suggested. A number of studies have documented an increase in viral load as viral clearance is reduced during bacterial co-infection. It is, however, unclear whether this is from bacterial and viral cooperation/interactions or simply from bacteria burdening the host immune system resulting in the reduction of viral eradication. Therefore further research is required to develop our understanding of the interaction between bacteria and viruses within co-infection (Peltola and McCullers, 2004; Iverson et al., 2011; Smith and McCullers, 2014).

## Conclusion

Viral infection aids bacterial infection in a number of ways, including unveiling/providing more sites for adhesion, impairing immune responses and causing cell and tissue destruction allowing for the spread of bacteria and development of invasive infection. Bacterial infection is then able to worsen clinical outcome and the severity of disease. Of course viral and bacterial co-infection can be mutually beneficial, further helping viral infection, which is bad news for public health. Although antibiotics can help reduce the impact of co/secondary bacterial infection, we still need to better understand the interactions between viruses, bacteria and their host, and to fully understand all mechanisms of disease. Particularly in light of increased antibiotic resistance and the ability of microbes to adapt and evade vaccine induced immunity.

The aim of this review was to emphasize the historical and continuing threat of influenza and to highlight the risk of bacterial co/secondary infection. Vaccines and antibiotics are readily available, however, with antibiotic resistance at an all-time high, vaccination is becoming even more vital in the fight against influenza epidemics and pandemics and the bacterial co/secondary infections commonly associated. It is important to examine the strains and types of bacteria and viruses being spread amongst and transmitted throughout the general public (or continue to in the case of influenza) to inform clinical treatment and development, particularly in the setting of an influenza epidemic or pandemic. As the threat from influenza is ever changing, we need to ensure we know what strains are circulating, which could cause issue and how they interact with other potential pathogens. This preparation also entails monitoring the changing epidemiology of bacterial pathogens associated with secondary infection, such as capsule switching which help *S. pneumoniae* evade immunity (Pai et al., 2005a,b).

## Author Contributions

DM, DC, and SC designed, planned and wrote the manuscript.

## Conflict of Interest Statement

SC acts as principal investigator for clinical trials and other studies sponsored by the University Hospital Southampton NHS Foundation Trust/University of Southampton that are funded by vaccine manufacturers but receives no personal payments from them. SC has also participated in advisory boards for vaccine manufacturers but receives no personal payments for this work. SC has received financial assistance from vaccine manufacturers to attend conferences. All grants and honoraria are paid into accounts within the University of Southampton, or to independent charities. DC was employed for 18 months on a GSK funded research project in 2014/15. The other author declares that the research was conducted in the absence of any commercial or financial relationships that could be construed as a potential conflict of interest.
